# A national survey of community rehabilitation service provision for people with long Covid in Scotland

**DOI:** 10.12688/f1000research.27894.2

**Published:** 2021-03-26

**Authors:** Edward Duncan, Kay Cooper, Julie Cowie, Lyndsay Alexander, Jacqui Morris, Jenny Preston

**Affiliations:** 1 Nursing, Midwifery and Allied Health Professions Research Unit, Faculty of Health Sciences and Sport, University of Stirling, Stirling, FK9 4LA, UK; 2 School of Health Sciences, Robert Gordon University, Aberdeen, AB10 7QG, UK; 3 School of Health Sciences, University of Dundee, Dundee, DD1 4HJ, UK; 4 Douglas Grant Rehabilitation Centre, Ayrshire Central Hospital, Ayr, KA12 8SS, UK

**Keywords:** COVID-19, long Covid, community rehabilitation, allied health professions, survey

## Abstract

**Background:** Over 50 million cases of COVID-19 have been confirmed globally as of November 2020. Evidence is rapidly emerging on the epidemiology of COVID-19, and its impact on individuals and potential burden on health services and society. Between 10–35% of people with COVID-19 may experience post-acute long Covid. This currently equates to between 8,129 and 28,453 people in Scotland. Some of these people will require rehabilitation to support their recovery. Currently, we do not know how to optimally configure community rehabilitation services for people with long Covid.

**Methods: **This national survey aimed to provide a detailed description of current community rehabilitation provision for people with long Covid in Scotland. We developed, piloted, and conducted a national electronic survey of current community rehabilitation service provision for people presenting with long Covid symptomatology. Our sample were the Allied Health Professions Directors of all 14 territorial NHS Health Boards in Scotland. Fixed response and narrative data were analysed descriptively.

**Results:** Responses were received from all respondents (14/14), enabling a national picture to be gained. Almost all Health Boards (13/14) currently deliver rehabilitation for people with long Covid within pre-existing services. ****Fatigue (11/14) and respiratory conditions (9/14) were the two most common presenting problems of patients. Most long Covid community rehabilitation services are delivered through a combination of face-to-face and digital contact (13/14).

**Conclusions:** Community rehabilitation for people with long Covid is an emerging reality. This survey provides a national picture of current community rehabilitation for people with long Covid. We do not know how community rehabilitation can be optimally delivered for this population. This is vital as community rehabilitation services were already under pressure prior to the emergence of COVID-19. Further research is urgently required to investigate the implementation, outcomes and cost-effectiveness of differing models of community rehabilitation for this patient population.

## Introduction

Since the initial emergency of COVID-19 in Wuhan, Hubei Province, China in December 2019, the disease has rapidly spread across the globe, with more than 50 million cases now confirmed globally (9 November 2020) ^1^. Among the consequences of COVID-19 is the impact of long Covid, where individuals are left with debilitating symptoms after the initial acute phase of infection ^2^. To date there is no agreed definition of what constitutes long Covid, however it has been proposed that it is when individuals have symptoms extending three weeks beyond onset (post-acute COVID-19) and 12 weeks beyond onset (chronic COVID-19) ^3^. Long Covid does not only affect people who were severely ill, but also people with milder symptoms, and those who were not hospitalised ^4^.

It is estimated that 10–35% of people infected with COVID-19 may experience post-acute long Covid ^3– 5^. Globally this equates to between five and 17.5 million people who may experience debilitating aftereffects of the infection. Within Scotland (as of 15 November 2020) 1,086,353 people have been tested for COVID-19 with 81,294 people testing positive. Using this data, we can estimate that between 8,129 and 28,453 people may have post-acute COVID-19, with around 812 people remaining significantly unwell at 12-weeks, commonly due to organ damage ^6^. Consequently, long Covid has received considerable media attention in Scotland and beyond, with action groups calling for more support for symptom management ^7^. As around 10% of people who experience mild COVID-19 may go on to experience long-term symptoms ^8^, it is important that rehabilitation is accessible to those presenting in community settings as well as being discharged from hospital.

Reported symptoms of long Covid vary widely. They commonly include respiratory, cardiopulmonary, neurological, musculoskeletal and mental wellbeing sequelae, as well as fatigue and loss of taste and smell ^4, 5, 9^. The presentation and severity of these symptoms are variable. Several people who have long Covid report a non-linear journey of recovery and describe their symptoms as moving around their body, such that as one symptom abates, another appears ^9^.

A currently unknown number of people with long Covid will require rehabilitation to support their recovery and increase their quality of life. As with other long-term conditions, rehabilitation for people with long Covid should be multidisciplinary, comprehensive, and tailored to individuals’ needs, in order to maximise function, quality of life and participation in society ^10^. Rehabilitation for long Covid is in its infancy. We do not currently know how rehabilitation can be optimally delivered for people with long Covid. Findings from a recent living systematic review found most publications have been expert opinion about how rehabilitation for long Covid should be delivered, indicating that high-quality research is required ^11^. Understanding how to optimally deliver long Covid community rehabilitation is vital, as rehabilitation services need to cope with additional demand while continuing to provide rehabilitation for other, often vulnerable, patient populations ^12^.

This paper reports a recently conducted national survey of current community rehabilitation provision for people with long Covid. The aim of the survey was to provide a detailed description of current community rehabilitation provision for people with long Covid across Scotland. We believe it to be the first published national survey describing long Covid rehabilitation models of practice. The survey is the first step in a programme of research to investigate how community rehabilitation can be optimally delivered for people experiencing long Covid.

## Methods

### Design

Using the Jisc online survey tool we developed and conducted a national electronic survey for the Directors of Allied Health Professions of all 14 territorial NHS Health Boards in Scotland. The aim of the survey was to discover their current service provision for rehabilitation of people presenting with long Covid symptomatology in the community. The survey is reported in keeping with recommended reporting guidance for surveys ^13^.

### Survey development

An initial draft survey was developed by the study authors. This incorporated fixed item and narrative response survey questions, informed by the TIDieR Intervention Description checklist ^14^:

- **How?** How is long Covid rehabilitation delivered in your board area?
- **Why?** What are the main problems that patients require rehabilitation for?
- **What is provided?** Please describe the service as fully as you can.
- **Who provides?** What professional groups are involved in delivering long Covid rehabilitation in your board area?
- **How/where is it provided?** How do patients access long Covid rehabilitation in your board area?
- **When and How much?** Can you describe the timing and duration of typical long Covid rehabilitation in your board area?

We conducted a small pilot of the initial survey content with the Scottish Government’s Professional Advisor for Rehabilitation, the National Clinical Lead for Digital Health and Care, the Allied Health Professions’ Improvement Advisor for Healthcare Improvement Scotland, and the director of services of Chest Heart and Stroke Scotland. Minor changes to the survey wording were made based on their feedback. A copy of the final questionnaire used in this study is available as extended data.

### Sample

Healthcare in Scotland is primarily delivered through NHS Scotland’s 14 territorial Health Boards. Each Health Board covers a separate region. Together they cover the entire Scottish population. They are responsible for the protection and improvement of the health of the people in their region and the delivery of healthcare services. Each Health Board has a Director of Allied Health Professions. We invited all 14 Directors of Allied Health Professions to participate in this survey via an emailed letter. To minimise the potential of attrition bias, the letter from the study authors containing a link to the survey was emailed by the Scottish Government’s Professional Adviser for Rehabilitation to each of the Directors.

### Data collection

The online survey was launched on 14 October 2020 and closed on 6 November 2020.

### Data analysis

Fixed response item data (Questions 1,2,4,5,6,8a,9) were analysed descriptively. Narrative responses to open ended questions (Questions 3a, 4a, 5a, 6a, 8ai) were mostly short statements and insufficient to conduct for a formal thematic analysis or other qualitative method. Instead two members of the research team (ED, KC) reviewed responses and descriptively report them in the paper where they related to the study results.

### Ethics

As the study surveyed current practice it did not require research ethics approval by the NHS. Data was stored on password protected University servers in compliance with European Union General Data Protection Regulation (GDPR) standards of data protection and storage. The covering letter to potential participants explained the reasons for the survey and that their anonymised responses may be published. Informed consent to participate and for the publication of results was implied through their return of the study questionnaire.

## Results

We received responses from all 14 Directors of Allied Health Professions, enabling a national picture of community rehabilitation service delivery for people with long Covid to be gained. An anonymised copy of all survey response data is available as underlying data.

### How is long Covid rehabilitation being delivered?

Almost all Health Boards (13/14) are currently delivering rehabilitation for people with long Covid within pre-existing services. One Health Board has developed a new service for people requiring long Covid rehabilitation, and another is currently developing a new service. Data on the numbers of patients who have received long Covid rehabilitation to date were not available from most respondents (12/14), indicating that routine rehabilitation data collection methods are not yet universally established. In services that were able to provide referral number data (2/14), one respondent (from a rural island locality) stated that they had received a referral for one patient in total, while the Health Board with a specialist long Covid service stated that they had received 95 referrals in eight weeks.

### What are the main problems that patients require rehabilitation for?

Respondents reported that the main symptoms requiring rehabilitation interventions were fatigue (11/14), respiratory conditions (9/14), musculoskeletal conditions (6/14), mental health (5/14), and neurological impairments (4/14). One respondent stated that patients who were referred to their service experienced fatigue (86%), respiratory symptoms (67%), reduced mobility/exercise tolerance (60%), low mood, anxiety, depression (43%), cardiac symptoms (24%), sleep disturbance (24%), and weight management concerns (12%).

### What does long Covid rehabilitation consist of?

Respondents did not describe the therapeutic content of long Covid rehabilitation in any detail, referring instead to the professions that were involved in delivery of the service (see below). One respondent described their service providing energy conservation advice and assessment of aids and adaptations. Another respondent described their service as providing fatigue management, confidence building, muscle strengthening, anxiety management, nutritional advice, breathing re-education, and activities to support individuals to regain function. Another said their service used a combination of pulmonary rehabilitation and community reablement. A final respondent described their service as providing individualised goal setting based on symptomatic presentation.

### Who provides long Covid rehabilitation?

Community rehabilitation service provision for people with long Covid is multidisciplinary. Almost all services (13/14) include occupational therapy and physiotherapy. Many include dietetics (11/14) and speech and language therapy (9/14). Half include psychology input (7/14). In addition, three services reported being able to refer to, or having the involvement of differing resources including post intensive treatment nursing teams, therapy assistant practitioners, outpatient services for people with neurological conditions, spiritual care teams, and specialist rehabilitation medical consultant services.

### How/where is community rehabilitation for people with long Covid provided?

Most long Covid rehabilitation services are delivered through a combination of face-to-face and digital contact (13/14). While precise numbers were not available, respondents reported large variations in the percentage of rehabilitation being delivered through the different forms of delivery, depending on clinical need. One respondent reported that their primary delivery route was digital. Another reported only delivering long Covid rehabilitation face-to-face, with no digital service.

### What is the timing (post COVID-19 diagnosis) and duration of typical long Covid rehabilitation?

Almost all respondents (13/14) reported patients being able to access long Covid rehabilitation through either hospital or GP referral. Many respondents stated that patients could also access long Covid rehabilitation through self-referral (11/14). Some respondents (3/14) stated other routes of access to long Covid rehabilitation including interdisciplinary referrals from other allied health professionals and social care, as well as referrals from informal carers. Responses on typical duration of rehabilitation were limited. Three respondents stated it was dependent on the individuals’ needs.

## Discussion

Despite some expert opinion that referral to community rehabilitation is not required for many people who have had COVID-19 ^15^, our findings demonstrate that community rehabilitation for people with long Covid is an emerging reality and is being provided across Scotland. Community rehabilitation for people with long Covid is currently being delivered predominantly by multidisciplinary teams of allied health professionals, with other specialists available as required. This is in keeping with community rehabilitation for other long-term conditions ^16^. We have found variation in the modes in which long Covid rehabilitation is currently being delivered (face to face/digital/mixed) and provided (integrated services/new services) in Scotland. Symptoms that people with long Covid are presenting with to rehabilitation services are in keeping with the literature to date and provide an indication of the skill-mix and expertise required within a long Covid rehabilitation service. Irrespective of the number of patients requiring rehabilitation for long Covid within a particular health board area, services will need to be able to provide appropriate and accessible rehabilitation, responsive to the diverse symptomatology and wider impact of the condition. Mode of delivery will be compounded by ongoing physical distancing measures.

This study has several limitations. We do not yet know which modes of delivery are most appropriate for this patient population. Innovative tele-health services are beginning to be proposed for this patient group ^17, 18^, and the one Health Board in our survey that reported developing a new long Covid specific service, described providing a predominantly digital service. Data on rehabilitation services in Scotland is not routinely collected, so detailed information on the numbers of referrals of people experiencing long Covid, the problems with which they were presenting, duration of rehabilitation, and specific interventions delivered was unavailable.

This survey provides a national picture of current community rehabilitation for people with long Covid symptomatology. To the best of our knowledge this is the first national survey of its kind. There is still lots to learn about current practice. While data on numbers of referrals, and content and duration of rehabilitation was requested, this information was not available to most of the respondents. A description of current services also does not provide any information on the effectiveness of the community rehabilitation service for people with diverse presentations, or of its perceived acceptability by its recipients. Detailed data on services and their recipients is vital and urgently required, to guide effective and efficient clinical practice and service planning and delivery. Following up this survey with qualitative interviews or a focus group would have provided richer and more in-depth information, however these options were not feasible within the timeframe that was available. Further research into long covid rehabilitation is now being conducted by the research team which includes interview and focus groups methods and we are confident that these methods will enhance our understanding of current community rehabilitation for people experience long covid.

Several UK bodies and individuals have published expert opinion recommending a stepped, needs-based community rehabilitation approach incorporating information provision, self-management support and specialist services as required. They also recommend that rehabilitation should be individualised, progressive and utilise digital solutions ^19– 21^. How to optimise delivery of community rehabilitation is unknown, but vital to determine, given that rehabilitation services need to cope with additional COVID-19 demand whilst continuing to provide rehabilitation for other, often vulnerable, patient populations ^21^.

Community rehabilitation is a complex intervention ^22^, which is provided in different ways according to clinical need, geographical location and financial costs. This complexity is further exacerbated when treating people with long Covid where the impact of the clinical sequalae is still unknown. While community rehabilitation has been routinely provided within the NHS in Scotland for many years, there are many unknowns regarding the delivery of community rehabilitation for people with long Covid. Therefore, research is urgently required to evaluate which models of community rehabilitation work, in what circumstances, and with whom.

## Conclusions

This paper reports the findings of a national survey of current community rehabilitation provision for people with long Covid in Scotland. Almost all current services are providing a community rehabilitation response within current service provision. There is variation in the way in which these services are provided. Some information was unavailable due to the lack of routine data collection. With growing numbers of people presenting with symptoms of long Covid, further research is urgently required to investigate the implementation, outcomes and cost-effectiveness of differing models of community rehabilitation for this patient population.

## Data availability

### Underlying data

DataSTORRE: Stirling Online Repository for Research Data. Survey Data for Long covid rehabilitation study. http://hdl.handle.net/11667/164

This project contains the following underlying data

-    Anonymised survey responses in .xlsx format

Data are available under the terms of the Creative Commons Attribution 4.0 International license (CC-BY 4.0).

### Extended data

DataSTORRE: Stirling Online Repository for Research Data. Survey Data for Long covid rehabilitation study. http://hdl.handle.net/11667/165

This project contains the following underlying data

-    A copy of the survey sent to participants in .pdf format

Data are available under the terms of the Creative Commons Attribution 4.0 International license (CC-BY 4.0).
