# Supplementary material for: A national survey of community rehabilitation service provision for people with long Covid in Scotland
Source: F1000Res. 2021 Mar 26;9:1416. Originally published 2020 Dec 7. [Version 2] doi: 10.12688/f1000research.27894.2 (PMC8063521; doi:10.12688/f1000research.27894.2)
Supplement: Peer review history

# Peer review: Reviewer response for version 2

**DOI:** 10.5256/f1000research.55553.r82270

**Published:** 2021-04-21

**Authors:** Suzanne McDonough

**Affiliations:** 1 Institute of Nursing and Health Research, School of Nursing, Ulster University, Newtownabbey, UK; 2 School of Physiotherapy, RCSI University of Medicine and Health Sciences, Dublin, Ireland

I am happy that the authors have addressed my comments, I have nothing further to add.

Is the work clearly and accurately presented and does it cite the current literature?

Yes

If applicable, is the statistical analysis and its interpretation appropriate?

Not applicable

Are all the source data underlying the results available to ensure full reproducibility?

Yes

Is the study design appropriate and is the work technically sound?

Yes

Are the conclusions drawn adequately supported by the results?

Yes

Are sufficient details of methods and analysis provided to allow replication by others?

Partly

Reviewer Expertise:

Rehabilitation

I confirm that I have read this submission and believe that I have an appropriate level of expertise to confirm that it is of an acceptable scientific standard.

# Peer review: Reviewer response for version 2

**DOI:** 10.5256/f1000research.55553.r82271

**Published:** 2021-03-29

**Authors:** Lynette Mackenzie

**Affiliations:** 1 Discipline of Occupational Therapy, The University of Sydney, Lidcombe, NSW, 2006, Australia

Nothing to add.

Is the work clearly and accurately presented and does it cite the current literature?

Yes

If applicable, is the statistical analysis and its interpretation appropriate?

Not applicable

Are all the source data underlying the results available to ensure full reproducibility?

Yes

Is the study design appropriate and is the work technically sound?

Yes

Are the conclusions drawn adequately supported by the results?

Yes

Are sufficient details of methods and analysis provided to allow replication by others?

Yes

Reviewer Expertise:

Community based occupational therapy with older people and people with chronic conditions.

I confirm that I have read this submission and believe that I have an appropriate level of expertise to confirm that it is of an acceptable scientific standard.

# Peer review: Reviewer response for version 1

**DOI:** 10.5256/f1000research.30846.r77654

**Published:** 2021-02-16

**Authors:** Lynette Mackenzie

**Affiliations:** 1 Discipline of Occupational Therapy, The University of Sydney, Lidcombe, NSW, 2006, Australia

I found this article an interesting and important read. I was wondering how a person with long COVID is identified - especially if their symptoms persist over 3 months after their COVID infection, and whether or not their access to rehabilitation would be dependent on them complaining of symptoms to someone who could refer such as their GP. This might mean that there are not the full numbers of people with long COVID who would be presenting to rehabilitation services which would limit the feedback that such services could provide in this survey.

 I was also interested that it was only the lead allied health people who were surveyed (n=14) since these people might not have a full idea of what services are actually provided at the coal face, being in more management positions. It would be interesting to send a similar survey to all community rehabilitation health professionals to get more detailed information. I'm sure the rural/urban differences would be interesting to look at with a larger sample. 

 Having said that, all these limitations were adequately addressed by the authors. I'm sure there will be further work done in this area.

Is the work clearly and accurately presented and does it cite the current literature?

Yes

If applicable, is the statistical analysis and its interpretation appropriate?

Not applicable

Are all the source data underlying the results available to ensure full reproducibility?

Yes

Is the study design appropriate and is the work technically sound?

Yes

Are the conclusions drawn adequately supported by the results?

Yes

Are sufficient details of methods and analysis provided to allow replication by others?

Yes

Reviewer Expertise:

Community based occupational therapy with older people and people with chronic conditions.

I confirm that I have read this submission and believe that I have an appropriate level of expertise to confirm that it is of an acceptable scientific standard.

# Response

**Published:** 2021-03-17

**Authors:** Edward Duncan

**Affiliations:** Nursing Midwifery and Allied Health Professions Research Unit, UK

Dear Dr MacKenzie,

 Many thanks for your review and positive feedback. In response to another reviewer we have added a comment in the limitations of our revised paper, which highlights that some of our future research will investigate the issues that you raise.

# Peer review: Reviewer response for version 1

**DOI:** 10.5256/f1000research.30846.r75829

**Published:** 2021-02-09

**Authors:** Suzanne McDonough

**Affiliations:** 1 Institute of Nursing and Health Research, School of Nursing, Ulster University, Newtownabbey, UK; 2 School of Physiotherapy, RCSI University of Medicine and Health Sciences, Dublin, Ireland

This is an important and timely piece of work which provides an overview, at a national level, of community based rehabilitation for people with long Covid, and key gaps in knowledge.

 Strengths of the work are the survey design i.e. using Surge guidelines for reporting and TIDIER as a structure for the questions with a combination of closed and open narrative questions; the team piloted the tool prior to use and involved key stakeholders in its development. It is a well written manuscript with use of key recent supporting references. There was an excellent overall response rate to the survey, and within the survey to the individual closed questions. The response to some open questions was good, and is absent for others. Overall this work is an important first step to inform service provision for managing people with long Covid.

**Items to consider addressing:**

- Was there a plan to collate the responses to the narrative questions? and if so please add this to your data analysis section.

**Please clarify in your methods:**

- Were there reminders to respondents to complete the questionnaire or a single invitation only?
- Did the team consider other ways of enhancing the responses to the narrative questions? Might their open approach be a limitation to their survey? Do they think further closed questions with options may have helped, or a qualitative interview/focus group follow up?
- In the discussion, first paragraph, you state that there was a wide variation in the numbers accessing services, this needs to be qualified as I understand from your results that this data was only available for 2/14 of the respondents so is largely unknown.

Is the work clearly and accurately presented and does it cite the current literature?

Yes

If applicable, is the statistical analysis and its interpretation appropriate?

Not applicable

Are all the source data underlying the results available to ensure full reproducibility?

Yes

Is the study design appropriate and is the work technically sound?

Yes

Are the conclusions drawn adequately supported by the results?

Yes

Are sufficient details of methods and analysis provided to allow replication by others?

Partly

Reviewer Expertise:

Rehabilitation

I confirm that I have read this submission and believe that I have an appropriate level of expertise to confirm that it is of an acceptable scientific standard, however I have significant reservations, as outlined above.

# Response

**Published:** 2021-03-17

**Authors:** Edward Duncan

**Affiliations:** Nursing Midwifery and Allied Health Professions Research Unit, UK

Dear Professor McDonough,

 Thank you for taking the time to review this paper and for your helpful comments. Our responses to your review are below. 

***Items to consider addressing:***

- *Was there a plan to collate the responses to the narrative questions? and if so please add this to your data analysis section.*

RESPONSE: Narrative responses were mostly short statements and insufficient to conduct for a formal thematic analysis or other qualitative method. Instead two members of the research team (ED, KC) reviewed responses and descriptively report them in the paper where they related to the study results. We have added this clarification to the methods section of our paper.

***Please clarify in your methods:***

- *Were there reminders to respondents to complete the questionnaire or a single invitation only?*

RESPONSE: As stated in the methods section, under "data analysis" - reminders were sent to non respondents after 1 week.

- *Did the team consider other ways of enhancing the responses to the narrative questions? Might their open approach be a limitation to their survey? Do they think further closed questions with options may have helped, or a qualitative interview/focus group follow up?*

RESPONSE: Following up this survey with qualitative interviews or a focus group would have provided richer and more in-depth information, however these options were not feasible within the timeframe that was available. Further research into long Covid rehabilitation is now being conducted by the research team which includes interview and focus groups methods and we are confident that these methods will enhance our understanding of current community rehabilitation for people experience long covid. We have added this clarification to the methods section of our paper.

- *In the discussion, first paragraph, you state that there was a wide variation in the numbers accessing services, this needs to be qualified as I understand from your results that this data was only available for 2/14 of the respondents so is largely unknown.*

RESPONSE: We have removed this sentence in response to your feedback.
